# Comparison of the diagnostic performance of Enterosystem 18R and the BD Phoenix automated identification and susceptibility testing system for identifying gram-negative Enterobacteria: a preliminary study

**DOI:** 10.1128/spectrum.02260-25

**Published:** 2025-11-20

**Authors:** Bwambale Jonani, Patricia Peace Nakitende, Daniella Nabakka, Herman Roman Bwire, Emmanuel Charles Kasule, John Bosco Mundaka

**Affiliations:** 1 Department of Immunology and Molecular Biology, School of Biomedical Sciences, College of Health Sciences, Makerere University 58588, Kampala, Uganda; 2 Department of Clinical Laboratory, Sebbi Hospital, Nansana, Uganda; 3 Department of Obstetrics and Gynaecology, Sebbi Hospital, Nansana, Uganda; Icahn School of Medicine at Mount Sinai, New York, New York, USA

**Keywords:** diagnostic accuracy, BD Phoenix automated identification system, urinary tract infections, diagnostic agreement, Enterobacteria identification, Enterosystem 18R

## LETTER

Resource-limited laboratories require affordable alternatives to automated bacterial identification systems, which cost approximately $30–$35 per test in low-resource settings. We evaluated the Enterosystem 18R (which costs approximately $7 per test) (1), a semi-automated biochemical identification system, against the BD Phoenix automated identification system (Model M50, panel NMIC/ID-104) using 20 pre-characterized Gram-negative, oxidase-negative enterobacterial isolates from clinical urine specimens.

Twenty blinded isolates were obtained from Makerere University’s Microbiology Laboratory, originally characterized using standard biochemical protocols and confirmed by Bruker Biotyper MALDI-TOF (Version 4.1.100, Bruker Daltonics, 2019). Isolates were tested using Enterosystem 18R at Sebbi Hospital and BD Phoenix at the reference laboratory. Agreement was assessed at multiple confidence thresholds (≥50%, ≥70%, ≥85%, and ≥90%) for the Enterosystem 18R’s on-panel organisms only.

BD Phoenix identified all 20 isolates (100%), of which 11 were on-panel organisms for the Enterosystem 18R. When comparing these 11 on-panel organisms, the agreement between the systems was 45.5% (κ = 0.32) at ≥50% confidence. The agreement improved to 71.4% (κ = 0.58) at ≥85% confidence, but this restricted the analysis to only 7 of the 11 on-panel organisms (Table 1). Critical misidentifications occurred even with high confidence, including *Klebsiella pneumoniae* identified as *Serratia plymuthica* (98% confidence) and *Acinetobacter baumannii* as *Yersinia pestis* (88% confidence).

**TABLE 1:** Agreement between BD Phoenix and Enterosystem 18R at different confidence thresholds

Confidence threshold (%)	No. of isolates	% agreement	95% CI (%)	Cohen’s Kappa (κ)
≥50	11	45.5	16.7–76.6	0.32
≥70	8	62.5	24.5–91.5	0.48
≥85	7	71.4	29.0–96.3	0.58
≥90	6	66.7	22.3–95.7	0.48

Difficulties in color interpretation in biochemical reactions significantly contributed to identification failures. Several reactions showed similar color transitions that were challenging to reliably differentiate, particularly the yellow-orange to red changes in the decarboxylation tests (wells 2–4, 7). These subjective color interpretations likely contributed to both identification failure and discordant results. Unlike automated optical reading, this semi-automated system relies on the subjective human interpretation of reaction color transitions, which can vary with operator experience and lighting conditions.

The 45% failure rate for on-panel organisms and poor diagnostic agreement suggests limited clinical utility despite cost advantages. Laboratories would require alternative identification methods for nearly half of the isolates, negating cost savings and perpetuating empirical antibiotic prescriptions without organism-specific guidance. The risk is that this would continue to perpetuate irrational antimicrobial prescriptions when reliable pathogen identification is impossible (2, 3).

This preliminary finding is limited by the small sample size (*n* = 20), single-site evaluation, and lack of a systematic inter-operator variability assessment. Larger multi-site validation studies with molecular adjudication of discordant results are needed to inform clinical implementation of the Enterosystem 18R microorganism identification system, especially in low-resource settings that are already constrained by a lack of microbiological diagnostic capability.

These preliminary findings indicate that Enterosystem 18R faces significant operational technical challenges that could limit its utility in resource-limited clinical settings. Future improvements to the system should prioritize minimizing the subjective reaction color interpretation by integrating an automated optical reader or alternative detection methods with distinct endpoints. Nevertheless, its affordability may still offer an interim solution to enhance microbial identification and support antimicrobial stewardship in low-resource settings.

## Data Availability

The data sets generated and analyzed in this study are available in the manuscript. Detailed identification results for all 20 isolates are available at https://doi.org/10.6084/m9.figshare.30460175.

## References

[1] Liofilchem. 2025. Enterosystem 18R: biochemical Identification of Gram-negative, oxidase-negative Enterobacteria. Liofilchem. Available from: https://www.liofilchem.com/solutions/clinical/microbial-identification/id-systems/id-ast-systems-pure-colony/enterosystem-18r. Retrieved 26 Jun 2025.

[2] Wi TE, Ndowa FJ, Ferreyra C, Kelly-Cirino C, Taylor MM, Toskin I, Kiarie J, Santesso N, Unemo M. 2019. Diagnosing sexually transmitted infections in resource-constrained settings: challenges and ways forward. J Int AIDS Soc 22:e25343. doi:10.1002/jia2.25343 31468679 PMC6715950

[3] AMR Review. 2015. Rapid diagnostics: stopping unnecessary use of antibiotics. The review on antimicrobial resistance. https://amr-review.org/sites/default/files/Paper-Rapid-Diagnostics-Stopping-Unnecessary-Prescription-Low-Res.pdf.

